# Optimization of IC Separation Based on Isocratic-to-Gradient Retention Modeling in Combination with Sequential Searching or Evolutionary Algorithm

**DOI:** 10.1155/2013/549729

**Published:** 2013-11-14

**Authors:** Šime Ukić, Marko Rogošić, Mirjana Novak, Ena Šimović, Vesna Tišler, Tomislav Bolanča

**Affiliations:** Faculty of Chemical Engineering and Technology, University of Zagreb, Marulićev trg 19, 10000 Zagreb, Croatia

## Abstract

Gradient ion chromatography was used for the separation of eight sugars: arabitol, cellobiose, fructose, fucose, lactulose, melibiose, N-acetyl-D-glucosamine, and raffinose. The separation method was optimized using a combination of simplex or genetic algorithm with the isocratic-to-gradient retention modeling. Both the simplex and genetic algorithms provided well separated chromatograms in a similar analysis time. However, the simplex methodology showed severe drawbacks when dealing with local minima. Thus the genetic algorithm methodology proved as a method of choice for gradient optimization in this case. All the calculated/predicted chromatograms were compared with the real sample data, showing more than a satisfactory agreement.

## 1. Introduction

Isocratic elution exhibits some advantages over the gradient one, such as greater simplicity, lower cost, simpler instrumentation, and no need of column reequilibration between consecutive injections [1]. However, gradient elution is becoming almost unavoidable in conventional liquid chromatography, including ion chromatography (IC). In gradient mode the elution strength usually increases during analysis, thus providing much narrower chromatographic peaks and significantly shorter analysis time. Such characteristics are favored in cases with multicomponent samples which span a wide retention range [2].

The implementation of gradient elution implies finding the suitable gradient program. The major criterion for optimal elution is a good resolution between analytes accompanied with an acceptable analysis time. Existence of a good model to predict the column output is a crucial item in optimization of chromatographic elution. IC development in the last several decades was accompanied with the increasing number of models, either theoretical or empirical, which can be used to predict or to explain experimental chromatograms [3–18]. Among numerous approaches, the retention isocratic-to-gradient (iso-to-grad) model [12, 16] appears to be particularly interesting. This model is primarily developed for systems with a single-competing eluent; it is based on the transfer of retention information from isocratic to gradient elution mode. 

The linear solvent strength model is the first and practically the most important model that describes the relation between component retention and concentration of competing ion for isocratic elution. This theoretical model, developed by Snyder et al. in 1979 [3], in its origin considers only electrostatic effects leading to ion exchange retention. Therefore, the presence of other mechanisms or occurrence of factors influencing the retention will result in deviations from the linear model. To overcome this problem some authors include an additional factor as a correction for nonlinearity [12, 19, 20] in IC and other LC techniques. Such empirically obtained polynomial models were shown to fit isocratic experimental data significantly better than the linear one. The selection of polynomial instead of linear model does not have significant influence on the calculation time with contemporary computers. Therefore the authors decided to describe the isocratic behavior with the better fitting model, that is, the polynomial dependence between the logarithm of retention coefficient, *k*, and concentration of competing ion in eluent, *c*:

(1) $$\begin{array}{c} \log k=a_{0}+a_{1}\log c+a_{2}\text{lo}{\text{g}}^{2}c. \end{array}$$

In principle only three isocratic experiments are sufficient for the determination of regression coefficients, *a*_0−2_, of the model described by (1).

The retention coefficient is calculated as

(2) $$\begin{array}{c} k=\frac{t_{R}-t_{M}}{t_{M}}, \end{array}$$

where *t*_*R*_ is a component retention time and *t*_*M*_ is the column's holdup time. For the ion chromatography case the column's hold-up time is equal to the retention time of unretained compounds, that is, column void time *t*_0_ [21].

On the other hand, gradient elution can be described generally by the integral elution equation

(3) $$\begin{array}{c} t_{0}=\int_{0}^{t_{R}-t_{0}}\frac{dt}{k\left[c\left(t\right)\right]}. \end{array}$$

The integral from (3) can be approximated with a sum of integrals over small time intervals. Inside those intervals the coefficient *k* can be assumed constant and can be calculated as an average of *k* values at the interval boundaries, obtained according to (1). In this way, isocratic retention information is transferred into gradient environment.

The iso-to-grad approach allows for the prediction of retention time for practically any gradient using only several isocratic experiments. Nevertheless, the optimization of gradient elution remains a severe and complex problem, especially knowing that the number of different gradient programs in any gradient domain is practically unlimited. The problem can be simplified by setting different constraints on the domain (i.e., defining the sets of finite intervals in which the gradient curve may change, finite sets of gradient curve shapes, or finite sets of allowed gradient slopes [22–25]). Unfortunately, the usage of any constraint involves the possibility of missing the true optimum. An increase of the number of allowable options inside each finite set will produce a finer description of the real experimental domain. However, it will at the same time increase significantly the number of possible gradient profiles. This leads to another problem, a domain of profiles that is too large substantially increases both the experimental effort and required modeling time, which may even exceed the performance of modern computers.

A mathematical model that is able to describe the optimization surface for gradient IC is generally a nonlinear and very complex function. Therefore it is not feasible to solve it (to find optimum) analytically. The fact that there are often multiple solutions (local extremes) complicates the situation even more. There are several classes of approaches available for solving this kind of problems among which are sequential searching procedures and evolutionary algorithms [26].

**Figure 1:**
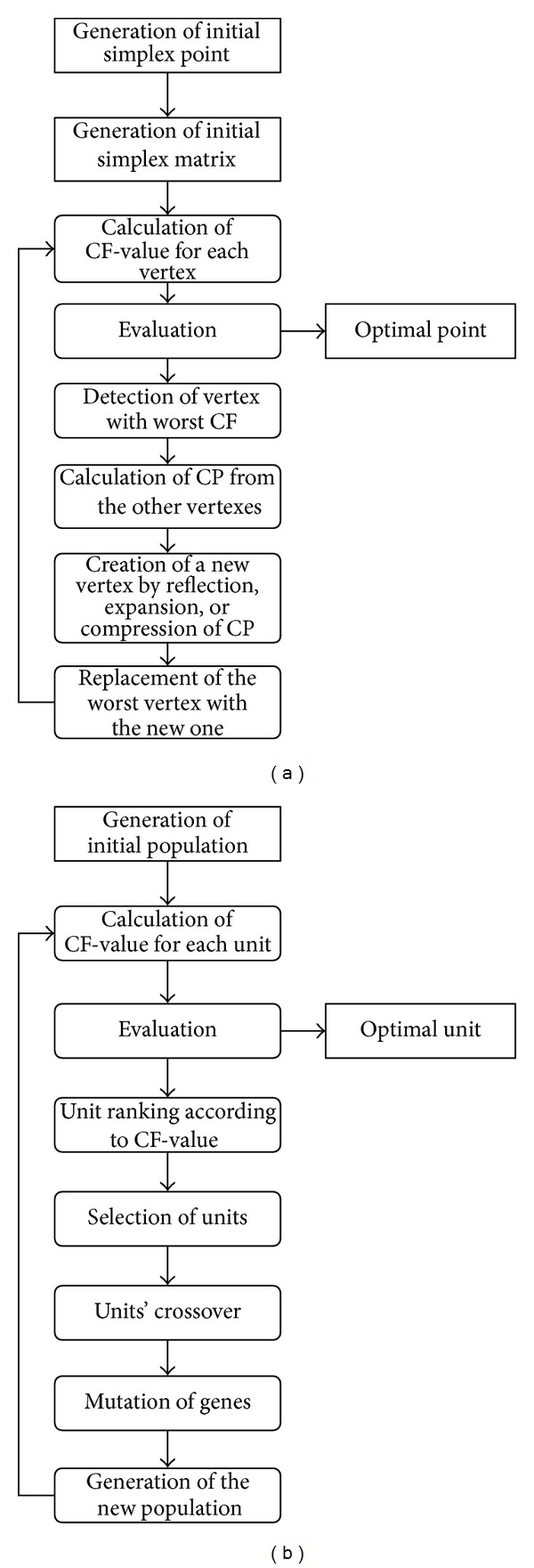
Block diagrams of (a) simplex and (b) genetic algorithm methodology. CF represents criterion function and CP is the centroid point of the hyperspace excluding the worst vertex.

One of the best known sequential methods is the Nelder-Mead simplex method [27]. The schematic block diagram of simplex methodology is presented in Figure 1(a). The user defines a very restricted number of simplex points, that is, vertexes, usually one more than the number of factors to be varied, *N*_*F*_. The function to be optimized is evaluated at the simplex vertexes, either experimentally or by calculation, using a previously constructed model. Then a decision is made about the worst vertex, which is replaced by a vertex that is reflected through the *N*_*F*_-dimensional surface (the reflection being defined by the rest *N*_*F*_ vertexes). According to the situation, the reflected vertex can be further expanded or contracted. Thus the next *N*_*F*_ + 1 simplex is created. Valuation and new simplex creation processes repeat until a predefined criterion is fulfilled.

Evolutionary algorithms mimic different biological processes in an attempt to optimize highly complex functions [28]. Genetic algorithms [29, 30] (Figure 1(b)), as a representative of the evolutionary algorithms, are based on genetic inheritance and Darwinian strive to survival [31]. In other words, they simulate the biological evolution. They allow a population composed of many individuals to evolve under specified selection rules to a state that optimizes the predefined objective function value [28].

This work is focused on the application of simplex methodology and genetic algorithm in the optimization of gradient elution in IC. The conventional application of these two methodologies in gradient IC implies the search for the optimum in the real experiment domain. A limited set of experiments is performed initially and, subsequently, the experiments with the worst output are replaced by new, better ones. This might take a long time and a lot of experimental effort. In the particular approach described by this paper, the number of experiments was practically minimized; the novel “experiments” to replace the old ones are not performed in the real domain, but by applying the initially constructed iso-to-grad model, that is, on a computer.

## 2. Materials and Methods

### 2.1. Instrumentation

The experiments were performed on a Dionex ICS-5000 (Thermo Fisher Scientific) ion chromatographic system, equipped with a dual pump (DP-5), eluent generator module (EG-5) with EGC III KOH cartridge, degas unit on eluent generator, continuously regenerated anion trap column (CR-ATC), thermostatically controlled detection module (DC-5), and an autosampler (AS-AP). For performing the separation, the system was equipped with a Dionex strong anion exchange column CarboPac PA20 (3 × 150 mm) and a respective guard column (3 × 30 mm). The detection mode was pulse amperometry, with reference electrode Ag/AgCl and working gold electrode. The separation was performed at constant temperature of 30°C, while the detection temperature was 20°C. The eluent flow rate was 0.5 mL/min, the sample loop volume was 10 *μ*L, and the data collection rate was 1 Hz. The whole system was computer controlled by the Chromeleon 7 software.

The simplex and genetic algorithm modeling required using a computer. We wrote the codes for both algorithms in the Matlab 2010b environment.

### 2.2. Reagents and Solutions

Standard solutions of 8 sugars: arabitol (150 ppm), cellobiose (600 ppm), fructose (1000 ppm), fucose (100 ppm), N-acetyl-D-glucosamine (500 ppm), lactulose (3000 ppm), melibiose (600 ppm), and raffinose (1000 ppm) were prepared by diluting appropriate amounts of solid compounds in deionized water (the first six sugars were from Sigma-Aldrich, USA, and the last two from Dr. Ehrenstorfer, Germany). The solid compounds were 97% or higher purity. Working mix solutions were prepared from standard solutions in concentrations 100 times lower than the standard ones. All solutions were preserved at 4°C. Working eluent solutions were hydroxide solutions prepared online in the eluent generator module by pumping water through the eluent generator chamber.

In all cases, 18 MΩcm^−1^ water (Millipore, USA) was used.

## 3. Modeling

### 3.1. Retention Modeling and Calculation of Resolution

In order to determine parameters of isocratic retention model (1) for each sugar, a set of 5 isocratic experiments was performed; the experiments were equidistantly distributed within the range from 2 to 98 mM KOH. Peak maximum retention was modeled as common; however, isocratic models were also defined for the 50% peak height point at the fronting side as well as for the 50% peak height point at the tailing side. These three retention times associated with each peak were used to predict the resolution between the analyzed sugars according to [32, 33]

(4) $$\begin{array}{c} R_{S}=1.18\frac{t_{R(2)}-t_{R(1)}}{w_{0.5(1)}+w_{0.5(2)}}, \end{array}$$

where *t*_*R*(1)_ and *t*_*R*(2)_ are retention times of two adjacent sugars and *w*_0.5(1)_ and *w*_0.5(2)_ are corresponding peak widths at half peak heights.

The void time needed for prediction of component retention under gradient elution was the same as in the isocratic case.

### 3.2. Criterion Function

Simplex vertexes or units from the genetic population were valued by the criterion function, CF:

(5) $$\begin{array}{c} \text{CF}={\left(\frac{1}{\sum R_{S}}\right)}^{\alpha }\cdot {t_{A}}^{\beta }. \end{array}$$

Two optimization goals were implemented into the criterion: the maximal sum of resolutions between eluted components ∑*R*_*S*_ [brackets in (5)] and the shortest analysis time *t*_*A*_. The desired balance between the goals was adjusted by the proper choice of criterion function weights *α* and *β*. A possible misbalance would produce an elution that is too long at one extreme or a peak overlapping at another. Therefore it was necessary to select the appropriate weight values. For the selection purpose both weights were varied from 1 to 5 using the step of 1.

### 3.3. Gradient Domain Scanning

The same gradient domain was searched for the optimal separation conditions with both the simplex and genetic algorithms. The eluent concentration was ranging from 5 to 95 mM KOH; only the gradients producing elution times shorter than 30 min were taken as the acceptable ones.

In the case of simplex approach, the acceptable time range of 30 min was split into *N*_*T*_ equidistant intervals (with varying *N*_*T*_). This provided *N*_*T*_ + 1 simplex factors (time points) in which the change of eluent concentration was possible. In the genetic algorithm case the equivalents to the simplex factors were termed genes. In the applied genetic algorithm, the 30-minute time range was split into 3 min intervals which provided 11 genes in which the concentration change was possible.

A linear gradient inside each of the intervals was presumed. The gradients were defined by eluent concentrations (the values of factors or genes) at start and end of each interval. For both applied approaches, the elution continued isocratically after the first 30 minutes, taking the last factor or gene value.

The optimization procedure implied searching for the optimal eluent concentration for each factor or gene. Therefore, an appropriate design in creation of initial matrix of points or units was needed.

### 3.4. Simplex Optimization

In the simplex optimization approach, the initial matrix of points was created using the Doehlert design [34, 35], which provides the mesh of lattice points uniformly and equidistantly distributed in the space around some central point [36]. If there are multiple optima in the searched domain, local optima (instead of the global optimum) will probably be reached using different initial matrices. To test this, we varied the parameters of the Doehlert design. These were (1) the number of simplex factors, (2) characteristic distance (concentration interval), and (3) central point position (central concentration). The number of simplex factors was varied from 1 to 5, using step 1. The concentration intervals were selected equidistantly within the predefined gradient domain range of 90 mM KOH to take values of 5, 10, 15, 30, 45, and 90 mM KOH. Central point was selected as an isocratic elution at a midpoint concentration within the selected concentration interval. For the three characteristic Nelder-Mead parameters, that is, coefficient of reflection, expansion, and contraction, standard values of 1, 2, and 0.5 were taken, respectively [37–39].

The optimization was set to terminate when the sum of absolute differences between the factor values of two consecutive iterations became lower than the predefined objective value of 10^−15^.

### 3.5. Genetic Algorithm Optimization

In the genetic algorithm approach, the first 30 minutes of elution were divided into 10 equally sized time intervals providing the set of 11 genes. The set of all genes is commonly termed the chromosome; it completely describes a unit with its peculiar elution characteristics.

The genetic populations were encoded by integer values. The initial population of 100 units was created by randomly assigning the concentration values from the predefined gradient domain range to all genes in the population. A rather low degree of elitism within the population units was set. This means that every unit from the population was valued according to (5); afterwards the worst 70% were removed. The rest of units were used as parents for the crossover procedure. The crossover was performed according to the rule: two parents-two descendants. We applied the uniform crossover procedure [28]; the genes forwarded from the first parent to the offspring were chosen randomly, and the rest of the genes came from the other parent. The new-born population was joined with the parents; afterwards such a created population was valued again. The worst 50% of units was removed and the rest became the new parent population. 

The mutation of genes is reality in every living population. Crossover without mutations restricts the characteristics of the new-born population from the characteristics of their parents; mutations bring new characteristics to the population. The percentage of mutations in new-born population may be considered an adjustable parameter. The number of mutations in new-born population was varied from 5 to 110 (using step 5) of overall 330 genes. The CF value (5) of the best unit was recorded after each crossover + mutation cycle. The optimization stopped after a predefined number of 200 cycles.

Since the random setting of initial population and application of mutations generally produces diverse results (different optima), a set of 10 consecutive runs was performed in all cases of genetic algorithm calculations.

### 3.6. Peak Shape Description

The generalized logistic distribution function was used for the description of peak shape of individual components in calculated chromatograms:

(6) $$\begin{array}{c} f\left(t\right)=\frac{C}{B}\cdot \frac{{\text{e}}^{(A-t)/B}}{{\left(1+{\text{e}}^{(A-t)/B}\right)}^{C+1}}. \end{array}$$

The median of distribution is associated with parameter *A*, parameter *B* characterizes the distribution width, and parameter *C* bears information about the distribution skewness [40, 41]. Peak description according to generalized logistic distribution function requires only four experimental (or calculated) data. These are the retention time of peak maximum, retention times of peak half-heights at fronting and tailing peak sides and the stretching factor, *S*_*F*_:

(7) $$\begin{array}{c} h\left(t\right)=S_{F}\cdot f\left(t\right). \end{array}$$

The stretching factor in fact equals the real chromatographic peak area [16].

## 4. Results and Discussion

Simplex and genetic algorithms were applied for the optimization of IC separation of the solution of 8 sugars. The conventional approach of simplex and genetic algorithms was improved by incorporating the iso-to-grad model [15]. Thus the simplex and genetic algorithms were allowed to search for the optimum in the virtual experiment domain, instead of the real experimental space, which produced the significant reduction of time and costs.

### 4.1. Isocratic Model Parameters

In principle, only three isocratic runs are needed to obtain coefficients of the quadratic dependence described by (1). Nevertheless, the higher number of isocratic experiments generally provides a more reliable isocratic model. In addition, it cannot be known *a priori* how the competing anion in eluent would affect the peak area and this is the feature essential for the peak shape prediction. Therefore we decided to perform 5 isocratic experiments. Where overlapping of the components was observed, working solutions of pure analytes were eluted as well. Thus a set of isocratic retention data was obtained to allow for the calculation of model parameters of (1). The calculated parameters are presented in Table 1. 

Apparently, the chosen polynomial model fits the retention behavior well for 7 of the 8 investigated sugars (*R* ^2^ ≥ 0.9989). In the case of arabitol there are some discrepancies. The observed error may be explained by comparing the retention times of all the 8 sugars at the lowest eluent concentration (2 mM KOH). The elution order observed was arabitol 1.53 min, fucose 3.85, N-acetyl-D-glucosamine 15.83, fructose 18.87, melibiose 23.98, raffinose 27.92, lactulose 40.12, and cellobiose 61.28 and void time was 0.93. In comparison to other sugars, the difference between arabitol elution time and void time is particularly small, only 0.60 min. It is important to recur that the data collection rate of the detector was only 1 Hz. Such a small rate could probably incorporate some error in the retention prediction, which would be especially noticeable for extremely fast eluting components like arabitol.

### 4.2. Investigated Gradient Domain

Some constrains were set on the investigated gradient domain in both applied optimization approaches. In the case of simplex optimization we had to limit the number of factors as well as the concentration interval investigated. These in turn determined the size of the simplex. Constrains of the genetic algorithm were posed by the number of genes (11) in population units as well as by the allowed concentration values for each gene (integer encoding of the population). Although the acceptable time of IC analysis was set to 30 min for both approaches, it should be pointed out that the procedure was allowed to predict the optimal separation with elution longer than 30 min. However, such events were then simply excluded from further considerations. As for the eluent concentration range, concentrations higher than 95 mM produced poor separation of the several least retained sugars; concentrations below 5 mM led to very long and economically unjustified elutions.

Although the negative gradients are quite uncommon in IC, since they deteriorate the baseline behavior, the authors did not exclude them from the simulation. In some cases, after reaching the satisfactory separation of a few first eluting components the negative gradient may, in principle, slow down the elution of the remaining components that would otherwise lead to overlapping of their peaks.

### 4.3. Criterion Function Weights

**Figure 2:**
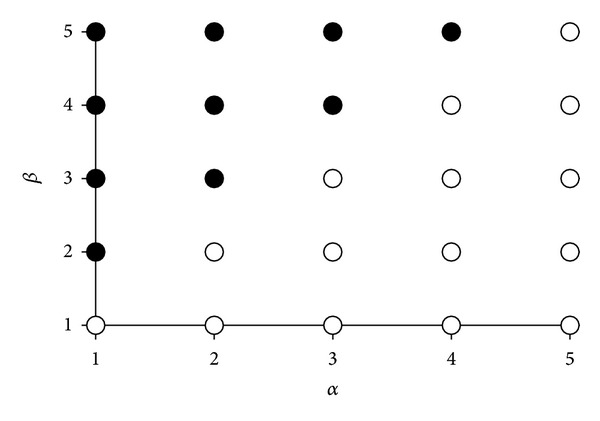
Selection of CF-function weights. Dark circles represent elutions with well separated peaks (all *R*_*S*_ ≥ 3) in acceptable analysis time (maximal 30 min) and the white ones are those with at least one overlapped peak pair (*R*_*S*_ < 3) or with exceeded analysis time.

As mentioned before, we had to assign the appropriate weights to the two contributions incorporated in the criterion function CF (5) that were selected for the valuation of simplex vertexes or genetic units. An appropriate balance between the weights should diminish the possibility of obtaining elution times that are too long or elutions with overlapped peaks. In addition, the simplex methodology always produces the same output for the same starting conditions, which is not the case in genetic approach due to mutations. Therefore, the selection of CF weights was performed according to the results obtained by simplex calculations; 25 simplex runs with different weight values were performed. The number of simplex factors was held constant at the value of 5 and the concentration interval was 15 mM KOH. The calculated optimal elutions were compared according to the existence of peak overlapping or analysis time that is too long. The peak overlapping threshold was set to *R*_*S*_ < 3, which is somewhat higher than the common values 1.5 or 2 [42]. We decided to set a more rigorous overlapping criterion since we were dealing with the retention models associated with possible errors. The obtained results, as shown in Figure 2, indicate that contribution of analysis time must be favored over the contribution of overlapping for the acceptable separation; that is, *β* must be higher than *α*. The unacceptable weight combinations are marked with hollow circles in Figure 2. We decided to select the combination of weight factors *α* = 1 and *β* = 2 for further calculations.

### 4.4. Simplex Optimization

**Figure 3:**
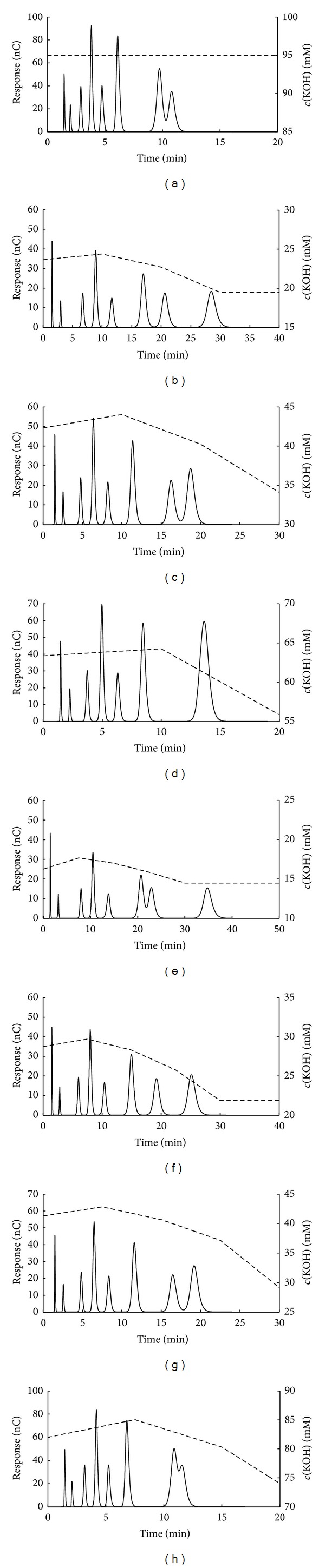
Searching for the appropriate number of simplex factors (*N*_*F*_) and concentration interval (Δ*c*). The above chromatograms were obtained for (a) *N*_*F*_ = 2 and 3 for all Δ*c*, *N*_*F*_ = 4 and Δ*c* = 30, 45, and 90 mM KOH, *N*_*F*_ = 5 and Δ*c* = 45 and 90 mM KOH, (b) *N*_*F*_ = 4 and Δ*c* = 5 mM KOH, (c) *N*_*F*_ = 4 and Δ*c* = 10 mM KOH, (d) *N*_*F*_ = 4 and Δ*c* = 15 mM KOH, (e) *N*_*F*_ = 5 and Δ*c* = 5 mM KOH, (f) *N*_*F*_ = 5 and Δ*c* = 10 mM KOH, (g) *N*_*F*_ = 5 and Δ*c* = 15 mM KOH, and (h) *N*_*F*_ = 5 and Δ*c* = 30 mM KOH.

The size of simplex was varied in the simplex optimization; 6 different concentration intervals and 5 different factor numbers were used. The characteristic results are shown in Figure 3. For the 2- and 3-factor cases the optimizations eventually reached the isocratic chromatograms, regardless of the size of the concentration interval applied (an example is presented in Figure 3(a)). In the case of 4-factor optimization, four different chromatograms were obtained depending on the starting point (Figures 3(a)–3(d)). The acceptable separation was obtained for the 5 mM concentration interval case, although the last sugar eluted at the edge of the tolerated period of 30 minutes (Figure 3(b)). With further increase of the concentration interval the analysis time decreased; at the same time the peak overlapping was observed (Figures 3(a), 3(c), and 3(d)). The results for the 5-factor optimization eventually produced five different chromatograms (Figures 3(a) and 3(e)–3(h)). The analysis time again decreased with the increase of the concentration interval. The smallest concentration interval provided the peak overlapping and analysis that is too long at the same time (Figure 3(e)). Good separations were predicted for the 10 and 15 mM concentration intervals (Figures 3(f) and 3(g)) with a distinction of significantly shorter analysis time for the 15 mM case. Further increase of the concentration interval produced peak overlapping (Figures 3(a) and 3(h)). Therefore, the best optimization result occurred when gradient domain was scanned by the algorithm characterized with 5 simplex factors and the concentration interval of 15 mM KOH as the starting point. It is important to notice that some different starting conditions produced significantly different “optimal” separations. This indicates that the applied simplex algorithm did not reach the global optimum in every case; that is, it had serious problems when dealing with local minima.

It can be noticed that gradients shown in Figure 3 continue even after the last component eluted from the column. Therefore it is necessary to clarify such observation to avoid any possible misunderstanding. The concentration and time domains were defined prior to the search for optimal separation conditions. Therefore, even for those cases where components eluted very fast, the remaining part of eluent concentration profile must exist (regardless of the fact that it has no influence on separation), but only as an artifact of the applied calculation procedure. The specific eluent concentration profiles shown in Figure 3 after the last eluted component are simply the first ones that the algorithm found. The selection of any other concentration profile continuing the last eluted component will have no effect on separation since it is already achieved.

### 4.5. Genetic Optimization

**Figure 4:**
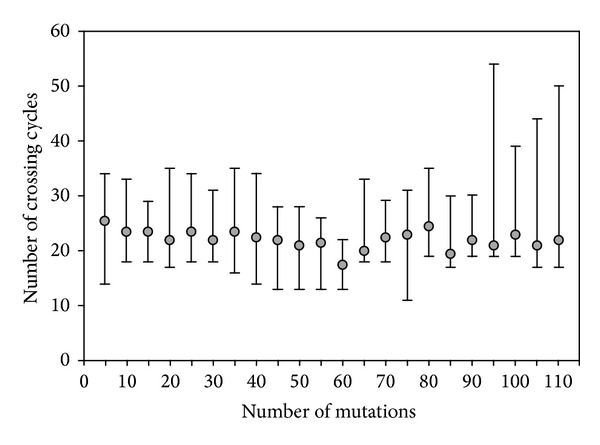
Relation between the threshold number of crossover cycles and number of mutated genes in offspring population; each population has a total number of 330 genes. Grey circles represent the average threshold number of crossover cycles among the 10 repeated calculations; black bars denote the scattering of 10 repeated calculations.

In the case of genetic algorithm, we were dealing with the percentage of mutations in new-born population and the number of crossover cycles as the important issues.  Figure 4 represents the results obtained for different number of mutations. 

Although the final number of crossover cycles was set to 200, the improvement of the best unit's CF value vanished normally after a much lower number of cycles. We termed it as the threshold number of cycles and we recorded it when the CF value dropped below 10^−12^. The gray circles in Figure 4 represent the median of the threshold number of cycles for 10 consecutive runs and the bars represent the corresponding span of values. According to the results plotted, the medians seem to be relatively independent of the number of mutations. However, considerably more scattering in the threshold number of cycles was observed when more than 90 genes were allowed to mutate. In conclusion, the crossover with 60 mutated genes (i.e., 18.2% of mutations) produced the smallest median of the threshold number of cycles (the shortest calculation time). At the same time it produced the minimum scattering of results. Therefore, this percentage of mutations was taken as the best one. Among the results calculated with this percentage of mutation, the gradient elution profile with the shortest elution time (18.89 min) was selected as the optimal one. It is important to point out that all 220 calculations using the genetic algorithm, regardless of the number of mutations applied or diverse initial populations randomly selected, produced extremely similar resolutions of adjacent components (*R*_*S*_ values, Table 2). The elution order of the analyzed sugars was always the same and none of the peaks overlapped. The standard deviation of predicted resolutions for all sugars and all 220 calculations was not higher than 1.28. Also, the standard deviation of maximal elution time was only 0.39.

### 4.6. Comparison of Calculated Optimal and Experimental Chromatograms

**Figure 5:**
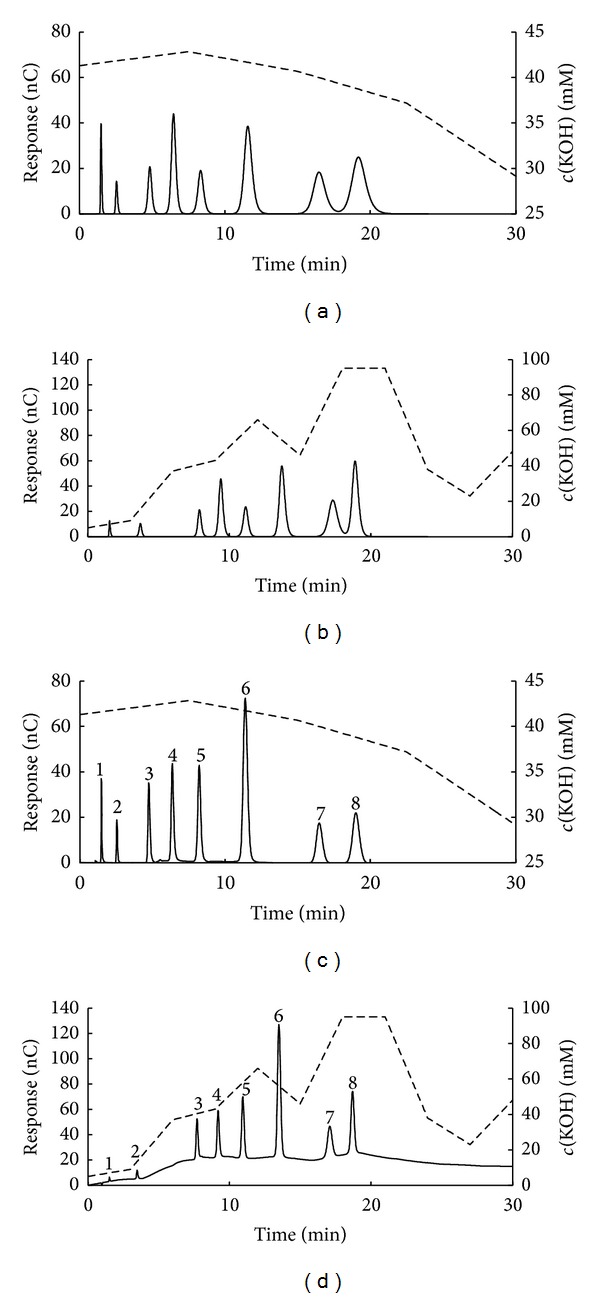
Comparison of experimental and predicted chromatograms obtained with optimal gradient profile: (a) predicted optimal separation by simplex algorithm, (b) predicted optimal separation by genetic algorithm, (c) real chromatogram corresponding to simplex optimization, and (d) real chromatogram corresponding to genetic algorithm. Dashed lines indicate the calculated optimal gradients. Analyzed sugars are marked according to elution order: (1) arabitol, (2) fucose, (3) N-acetyl-D-glucosamine, (4) fructose, (5) melibiose, (6) lactulose, (7) raffinose, and (8) cellobiose.

**Figure 6:**
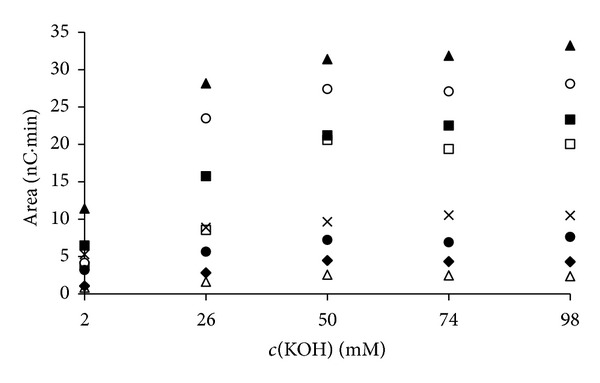
Peak areas for five isocratic experiments. The components are marked as follows: arabitol (*◆*), cellobiose (▲), fructose (□), fucose (△), lactulose (○), melibiose (×), N-acetyl-D-glucosamine (•), and raffinose (■).

In order to test the results obtained by the simplex and genetic algorithm optimization, the real IC analyses were performed under the calculated optimal KOH gradients (Figure 5). The experimental chromatograms are compared with the predicted ones. However, for the full description of predicted chromatogram one has to choose an appropriate distribution function to model the peak shape. The generalized logistic function proved a good choice for the peak shape prediction of inorganic anions in IC [16, 25] due to its simplicity and fair representation of experimental chromatograms. Therefore, in order to create a better presentation of the separated sugars, it was applied in this case as well. All the data needed for calculation of *A*, *B*, and *C* parameters of the generalized logistic distribution function are presented in Table 1, and the calculation procedure is described elsewhere [16, 25]. Therefore, only the stretching factor of the generalized logistic distribution function (7) will be discussed here. As mentioned before the stretching factor in this case equals the peak area of a real chromatogram. As said before, in this research the amperometric detector was applied. Since this detector is a concentration selective detector, an influence of competing anion concentration on the peak area is observed (Figure 6). For all the analyzed sugars a decrease of peak area with the increase of eluent concentration is observed. Isocratic experimental data were used to estimate the stretching factor, that is, peak area. Since the stretching factor depends on the competing anion concentration, the appropriate values for every peak were calculated in the following manner. First, peak retention time is identified. Second, competing anion concentration at that retention time is detected from the gradient profile calculated. Third, peak area is estimated at that competing anion concentration using the nearby experimental values from Figure 6 and linear interpolation.

The predicted and real chromatograms are compared in Figure 5. The predicted chromatograms have much broader bandwidths in comparison to the experimental ones, offering slight apparent overlapping of the two last eluted components (Figures 5(a) and 5(b)). Such overlapping was not a consequence of invalid optimization (all the calculated *R*_*S*_ values were higher than 3) but simply the product of the chosen peak-shape function, which seems to be less adequate for sugars than it was for inorganic anions. Despite that, the chromatograms generally match each other, confirming the applicability of both methodologies in the optimization of IC separation. Although the genetic algorithm may be considered somewhat better with respect to the analysis time (the predicted retention of the last eluting sugar of 18.98 min and 19.19 min for genetic and simplex optimization, resp.), it is obvious that both approaches provide similar analysis time. In both cases the last two eluted sugars (raffinose and cellobiose) have very close retention times. Their predicted resolutions are significantly smaller than for the other adjacent sugar pairs and almost equal to the predefined threshold value of *R*_*S*_ = 3. The required separation of these two sugars is, therefore, the most probable reason of such a long calculated analysis time.

## 5. Conclusions

This work describes the application of the two methodologies, that is, simplex and genetic algorithms, combined with the iso-to-grad retention model in the optimization of gradient IC separation. This combination allowed the finding of the optimum gradient profile in the virtual domain, thus minimizing the experimental effort and costs. The CF function weight factors were selected to favor the analysis time contribution (*α* = 1 for the resolution and *β* = 2 for the analysis time). The optimal gradient profile for the simplex approach was obtained using the 5 simplex factors and the concentration interval of 15 mM KOH in initiating the search. In the case of genetic algorithm, 18.2% of mutations in each offspring population were found to be the optimal percentage in fast and reliable finding of the optimum profile. The simplex methodology exhibited problems when dealing with local minima; that is, different starting conditions resulted in significantly different component elution times. At the same time, the genetic algorithm did not suffer from the local minima problem: 220 calculations provided 220 almost identical separations. The real sample experimental chromatograms obtained by applying the calculated optimal gradient profiles were compared with the predicted ones and good agreement was found. Both simplex and genetic algorithms in combination with iso-to-grad model proved a potential to be applied in gradient IC optimization. However, the genetic algorithm was coping better with the local minima problem. The developed approach offers a significant reduction of the experimental effort (only 5 isocratic experiments needed + few more for the overlapping peaks). In addition, there is no practical limitation on the gradient profile to be tested. Based on the results of this study, the genetic algorithm in combination with the iso-to-grad retention modeling may be recommended as the method of choice for optimization in gradient ion chromatography.

## Figures and Tables

**Table 1:** Parameters of isocratic retention models described by (1). The eluent concentration is expressed as mM.

	50% peak height at fronting side				Peak maximum				50% peak height at tailing side			
	*a*_0_	*a*_1_	*a*_2_	*R* ^2^	*a*_0_	*a*_1_	*a*_2_	*R* ^2^	*a*_0_	*a*_1_	*a*_2_	*R* ^2^
Arabitol	−0.201	−0.009	−0.031	0.9580	−0.188	−0.007	−0.030	0.9723	−0.153	−0.020	−0.027	0.9689
Cellobiose	1.808	0.127	−0.286	0.9999	1.814	0.125	−0.285	0.9999	1.820	0.126	−0.285	0.9999
Fructose	1.224	0.128	−0.261	0.9999	1.230	0.128	−0.261	0.9999	1.237	0.124	−0.259	1.0000
Fucose	0.353	0.282	0.234	1.0000	0.363	0.278	−0.231	1.0000	0.374	0.274	−0.228	1.0000
Lactulose	1.552	0.152	−0.291	1.0000	1.558	0.152	−0.291	1.0000	1.566	0.149	−0.289	1.0000
Melibiose	1.298	0.205	−0.288	1.0000	1.304	0.204	−0.288	1.0000	1.311	0.201	−0.286	1.0000
N-Acetyl-D-glucosamine	1.168	0.036	−0.243	0.9998	1.177	0.028	−0.239	0.9998	1.185	0.028	−0.238	0.9998
Raffinose	1.327	0.346	−0.262	0.9989	1.335	0.345	−0.261	0.9989	1.343	0.341	−0.260	0.9989

**Table 2:** Comparison of the calculated resolutions of 220 calculations using genetic algorithm.

	Minimal value	Maximal value	Standard deviation
Resolution			
Arabitol and fucose	11.98	15.75	0.61
Fucose and N-acetyl-D-glucosamine	18.30	24.93	1.28
N-Acetyl-D-glucosamine and fructose	4.64	5.83	0.25
Fructose and melibiose	4.95	5.81	0.15
Melibiose and lactulose	5.79	11.31	0.81
Lactulose and raffinose	4.71	9.77	0.62
Raffinose and cellobiose	3.00	3.68	0.13
Maximal elution/min	18.98	20.76	0.39
